# Influence of monoclonal anti-Lewis b, anti-H type 1, and anti-sialyl Lewis x antibodies on binding of *Helicobacter pylori* to MUC1 mucin

**DOI:** 10.1007/s11010-013-1833-1

**Published:** 2013-10-06

**Authors:** I. Radziejewska, K. Leszczyńska, M. Borzym-Kluczyk

**Affiliations:** 1 Department of Medical Chemistry, Medical University of Białystok, ul Mickiewicza 2a, 15-230 Białystok 8, Poland; 2 Department of Microbiology, Medical University of Białystok, ul. Mickiewicza 2c, 15-222 Białystok, Poland; 3 Department of Pharmaceutical Biochemistry, Medical University of Białystok, ul. Mickiewicza 2a, 15-230 Białystok 8, Poland

**Keywords:** Anti-Lewis b, Anti-H type 1, Anti-sialyl Lewis x, *Helicobacter pylori*, MUC1

## Abstract

To assess the influence of monoclonal anti-Lewis b, anti-H type 1, and anti-sialyl Lewis x addition on interactions of sugar structures of MUC1 mucin with *Helicobacter pylori.* The investigations were carried out on gastric juices of 11 patients and 12 *H. pylori* strains. The levels of Lewis b and sialyl Lewis x antigens on MUC1 were assessed by sandwich ELISA tests. Anti-Lewis b, anti-H type 1 or anti-sialyl Lewis x monoclonal antibodies were added to MUC1 to determine whether the adhesion activities of *H. pylori* isolates to examined mucin would be affected. Binding of bacteria to MUC1 was assessed by ELISA test. Clear inhibitory effect of examined antibodies was revealed in 6 of 12 examined *H. pylori* isolates independently on *babA2* status. In the rest of strains this effect was negligible. We confirmed participation of Lewis b, H type 1 and also sialyl Lewis x of MUC1 mucin in interactions with *H. pylori* independently on *babA* genopositivity. Not full inhibition and a lack of this effect in some strains suggest an existence of other mechanisms of *H. pylori* adherence to mucin.

## Introduction

*Helicobacter pylori* colonizes the gastric mucosa of more than half of the world’s population and is responsible for gastroduodenal diseases such as chronic gastritis, gastric and duodenal ulcers, and also gastric malignances [1–3]. It is interesting that most infected individuals do not reveal any clinical symptoms [4]. Bacterial virulence factors and host susceptibility features play a role in the development of infection. *H. pylori* colonizes the gastric mucosa by adhering to the mucous epithelial cells and the mucous layer lining the epithelium [4, 5]. To adhere, the bacterium uses adhesins responsible for recognizing of the specific carbohydrate structures. The best defined adhesins are the blood group-binding adhesin (BabA) with affinity to Lewis b and H type 1 antigens and sialic acid-binding adhesin (SabA) that binds sialyl Lewis x structure [6, 7]. Human Lewis antigens represent terminal modifications on mucins which are the main components of mucus and may mediate the attachment of *H. pylori* to the gastric mucosa. Expression of sialyl Lewis x in gastric mucosa is much increased in inflammatory state [4, 5, 8]. It is interesting that Lewis blood group antigens are also expressed on the O-specific chain of the lipopolysaccharide (LPS) of *H. pylori*. This can be understood as a kind of molecular mimicry between bacteria and host and could be involved in colonization process [9, 10].

There are three mucins, two secretory MUC5AC and MUC6, and membrane bound MUC1, which dominate in gastric mucus [4, 11–14]. Binding of Lewis b antigen on MUC5AC with BabA adhesin of *H. pylori* is considered as a major interaction occurring between bacterium and mucins [11, 15, 16]. The significance of the involvement of epithelial MUC1 mucin in the infection development is still under consideration. This mucin, the most highly expressed cell surface mucin in the stomach [17], seems to be important especially because a possibility to initiate an intracellular signaling in a response to *H. pylori* attachment [11, 15, 18]. As a consequence, extracellular domain of MUC1, together with attached bacterium can be detached from the cell surface. In this way MUC1 could limit, to some degree, development of disease ensuing from chronic *H. pylori* infection [11, 15]. Exact carbohydrate structures of MUC1 and *H. pylori* adhesins involved in binding of bacteria with this mucin are constantly under thorough examination.

In our study we decided to check possible involvement of Lewis b, H type 1 and sialyl Lewis x of MUC1 in adhesion to *H. pylori.* To study this, we used monoclonal antibodies to block suggested bindings.

## Materials and methods

### Patients and specimens

Eleven *H. pylori* infected patients with duodenal ulcers hospitalized in the Department of Medicine and Gastroenterology of Regional Hospital of Białystok, Poland, were included in the study. The patients were treated for 2 weeks with oral administration of omeprazole (2 × 20 mg per day), amoxiciline (2 × 100 mg), and tynidazole (2 × 500 mg). All the subjects were on a standard hospital diet served for the peptic ulcer patients. The tested gastric juices were taken on 11–13 day of the successful treatment. The presence of the bacterium was examined histopathologically and by urease test with gastric cells scraped under endoscopic examination.

To obtain high molecular mass material, the juices were chromatographed on a Sepharose 4B column as described before [19]. Concentrated material of the void volume was subjected to further analysis. The protein content was measured using bicinchoninic acid [20]. Samples of juices were diluted to the same protein concentration (0.005 mg/mL) prior to ELISA tests.

### Bacterial strains and culture conditions

*Helicobacter pylori* strains were isolated from gastric epithelial cells scraped from 12 individuals suffering from gastritis. The scrapings were collected before the beginning of the treatment, under endoscopic examination, from the prepyloric area and the body of the stomach. Immediately the scrapings were carried into the transport medium *Portagerm pylori* (bioMerieux, France). After homogenization, the bacteria were cultured on Pylori Agar and Columbia Agar supplemented with 5 % sheep blood (bioMerieux, France) for 7 days at 37 °C under microaerophilic conditions using Genbag microaer (bioMerieux, France). Microorganisms were identified upon the colony morphology, by the Gram method; the activity of the bacterial urease, catalase and oxidase were also determined. To prove *H. pylori* species, ELISA test (HpAg48; EQUIPAR, Spain) was used. Then the bacteria were subcultured in the same conditions and suspended at 1.2 × 10^9^ bacteria/mL in PBS.

### PCR and primers for *babA2* genotypes

DNA isolation from all examined bacterial strains was performed using QuickExtract™ Bacterial DNA Extraction Kit and was manufactured according to protocol. Extracted DNA from each strain was subjected to PCR for amplification of the *babA2* genes, applying one pair of primers (bab7-F: CCA AAC GAA ACA AAA AGC GT, corresponding to bp 105–124 of AF033654; bab7-R: GCT TGT GTA AAA GCC GTC GT, corresponding to bp 357–375 of AF033654) [8].

### Determination of Lewis b, H type 1 and sialyl Lewis x on *H. pylori*

Suspensions of *H. pylori* were diluted 50 times to get 2.4 × 10^7^ cells/mL and 50 μL of each isolate were coated onto microtiter plates (NUNC F96; Maxisorp, Roskilde, Denmark) and incubated at 37 °C overnight. The plates were washed three times (100 μL) in PBS, 0.05 % Tween (PBS-T; washing buffer) between all ensuring steps. Unbound sites were blocked with 100 μL of 1 % blocking reagent for ELISA (Roche Diagnostics, Mannheim, Germany) for 1 h. Then the plates were incubated with primary antibodies (anti-Lewis b, anti-H type 1 (both IgG class), and anti-sialyl Lewis x (IgM); for specifications of antibodies used in the study see Table 1) diluted 1:200 in 1 % bovine serum albumin (BSA) in washing buffer for 1 h. Then the plates were incubated with secondary antibody, horseradish peroxidase-conjugated rabbit anti-mouse IgG (for anti-Lewis b and anti-H type 1) or anti-mouse IgM (for anti-sialyl Lewis x) diluted (1:2,000) in the above buffer for 1 h. After washing four times in PBS, the coloured reaction was developed by incubation with 2,2′-azino-bis(3-ethylbenzthiazoline-6-sulfonic acid) (ABTS)—liquid substrate for horseradish peroxidase (Sigma, St. Luis, MO, USA). Absorbance at 405 nm was measured after about 30–40 min.

**Table 1:** Source of antibodies used in this study

Antibody	Clone	Source
Anti-MUC 1 (IgG)	BC2	Thermo scientific
Anti-Lewis b (IgG)	LWB01	Thermo scientific
Anti-Lewis b (IgM)	T218	Thermo scientific
Anti-H type 1 (IgG)	17-206	Abcam
Anti-sialyl Lewis x (IgM)	MAB2096	Millipore
Anti-*H. pylori* (polyclonal, biotin-conjugated)		Abcam
Anti-mouse IgG peroxidase conjugated		Sigma
Anti-mouse IgM peroxidase conjugated		Sigma

### Assessment of Lewis b and sialyl Lewis x on MUC1

MUC1 mucin from the void volume of a Sepharose 4B column of the each patient was analyzed for Lewis b and sialyl Lewis x content. To capture the examined mucin the anti-MUC1 monoclonal antibody BC2 (IgG) (diluted in 0.1 M bicarbonate buffer, pH 9.5 to final concentration 0.5 μg/mL) was coated (100 μL/well) onto microtiter plates overnight at 4 °C. The microtiter plates were washed and blocked as described above. Aliquots (50 μL) of the samples—high molecular mass material from the void volume after gel filtration on Sepharose 4B (containing 0.005 mg of protein/mL) were coated onto microtiter plates at room temperature for 2 h. After washing the plates were incubated with primary antibodies (anti-Lewis b and anti-siayl Lewis x (both IgM class) diluted (1:200) in 1 % BSA in washing buffer) for 1 h. After incubation with secondary antibodies the colored reaction was developed as described above.

### Binding of *H. pylori* to MUC1 mucin

The anti-MUC1 monoclonal antibody BC2 (diluted as mentioned above) was coated (100 μL/well) onto microtiter plates overnight at 4 °C. The microtiter plates were washed and blocked as described above. The plates were incubated for 2 h with aliquots (50 μL; 5 μg of protein/mL) of gastric juices at room temperature. Then, monoclonal anti-Lewis b (IgG), anti-H type 1 (IgG), and anti-sialyl Lewis x (IgM) (diluted 1:500 in washing buffer with 1 % BSA) were added to the wells. Wells without monoclonal antibodies added served as controls. The bacteria were diluted 50 times with PBS (to get 2.4 × 10^7^ cells/mL) and 100 μL of each strain were transferred onto microtiter plates which were incubated at 37 °C overnight. Then each well was treated with anti-*H. pylori* polyclonal, biotin-conjugated antibody (diluted 1:2,000 in washing buffer with 1 % BSA) at room temperature for 1 h. After being incubated with horseradish peroxidase-conjugated avidin D (Vector, Burlingame, CA, USA) (1:2,500), the colored reaction was developed as described above. The absorbance was recorded at 405 nm after 15–30 min.

### Statistics

Binding of *H. pylori* to MUC1 mucin (pretreated or not with anti-Lewis b, anti-H type 1, or anti-sialyl Lewis x) was subjected to statistical analysis (by STATISTICA 10.1 StatSoft program). Two-sided *t* test and Wilcoxon matched pairs test were used. Statistical significance was assumed at *p* < 0.05. Correlations of two variables (the relative amounts of Lewis b and sialyl Lewis x on MUC1 mucin versus the level of *H. pylori* bound to MUC1 pretreated or not with anti-Lewis b or anti-sialyl Lewis x) were calculated.

This study was approved by the Institutional Ethical Committee with the principles of the Declaration of Helsinki and informed consent was obtained from all patients.

## Results

Nine of 12 (75 %) of *H. pylori* isolates had a positive *babA2* genotype by PCR, applying the pair of primers used by Sheu et al. [8]. 271-bp PCR products were obtained what was electrophoretically confirmed (data not shown).

**Fig. 1:**
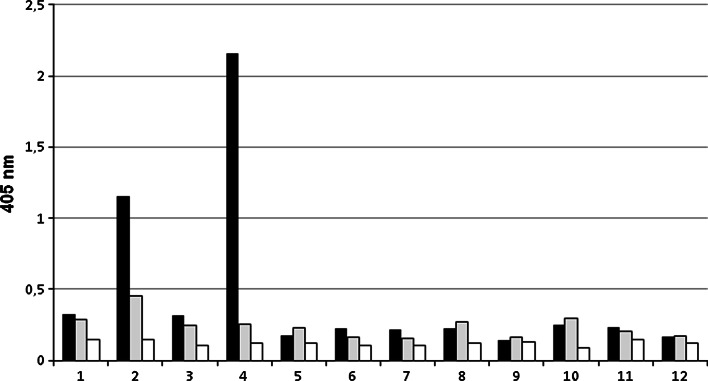
Relative levels of Lewis b (*black bars*), H type 1 (*grey bars*), and sialyl Lewis x (*white bars*) (as absorbance at 405 nm after reactivity with proper monoclonal antibodies) on *H. pylori* strains. *1*, *2*, *3*…—*H. pylori* isolates numbers

Expression of Lewis b, H type 1, and sialyl Lewis x antigens on *H. pylori* was detected by ELISA. Antigens expressions were determined as OD values (405 nm). For Lewis b OD ranged between 0.142 and 2.16. Two strains had high OD values of >1.5. OD for H type 1 antigen ranged from 0.152 to 0.452. The lowest values were revealed for sialyl Lewis x and ranged between 0.091 and 0.143 (Fig. 1).

**Fig. 2:**
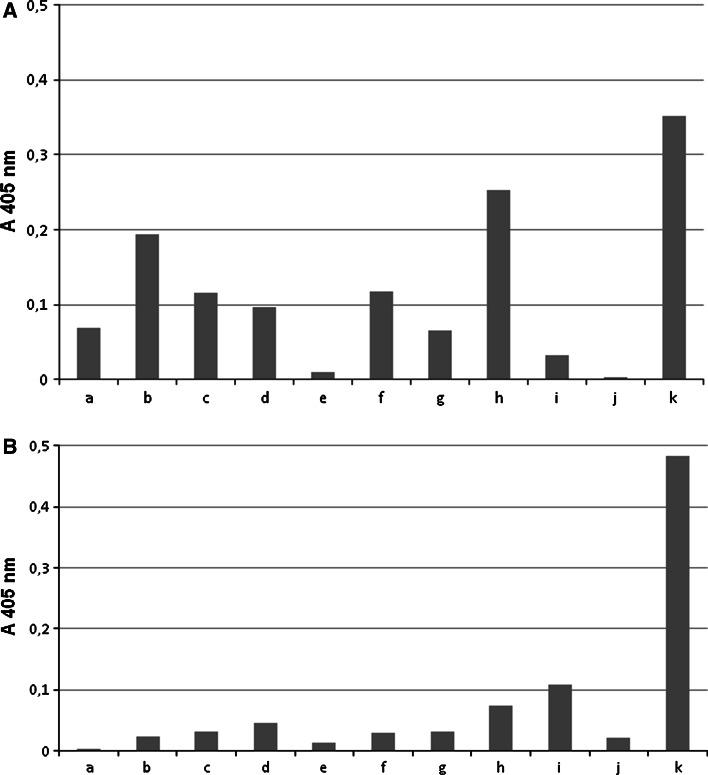
Relative levels of Lewis b (**a**) and sialyl Lewis x (**b**) antigens (as absorbance at 405 nm after reactivity with anti-Lewis b and anti-sialyl Lewis x antibodies) on MUC1 mucin (*n* = 11) selectively captured by anti-MUC1 antibody. *a*, *b*, *c*…—patients

Lewis b and sialyl Lewis x expression on MUC1 mucin from gastric juices of 11 patients were detected by sandwich ELISA. As capture antibody anti-MUC1 IgG class immunoglobulin was used, while as detection antibodies anti-Lewis b and anti-sialyl Lewis x IgM immunoglobulins were applied. Antigens expression was determined by the OD value (405 nm) and ranged between 0.003 and 0.352 (mean 0.119) for Lewis b structure (Fig. 2a) and between 0.003 and 0.483 (mean 0.079) for sialyl Lewis x (Fig. 2b).

**Fig. 3:**
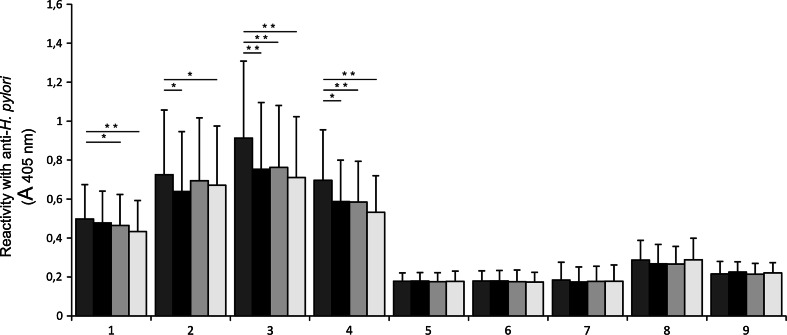
A *babA2*-positive *H. pylori* binding to MUC1 mucin of gastric juices (*n* = 11) selectively captured by anti-MUC1 antibody. MUC1 was not treated (*dark grey bars*), treated with anti-Lewis b (*black bars*), anti-H type 1 (*light grey bars*), or anti-sialyl Lewis x (*white bars*) before addition of *H. pylori* isolates. *1*, *2*, *3*…—*babA2*-positive *H. pylori* isolates numbers. *Bars* represent ± SD; * *p* < 0.05; ** *p* < 0.001

**Fig. 4:**
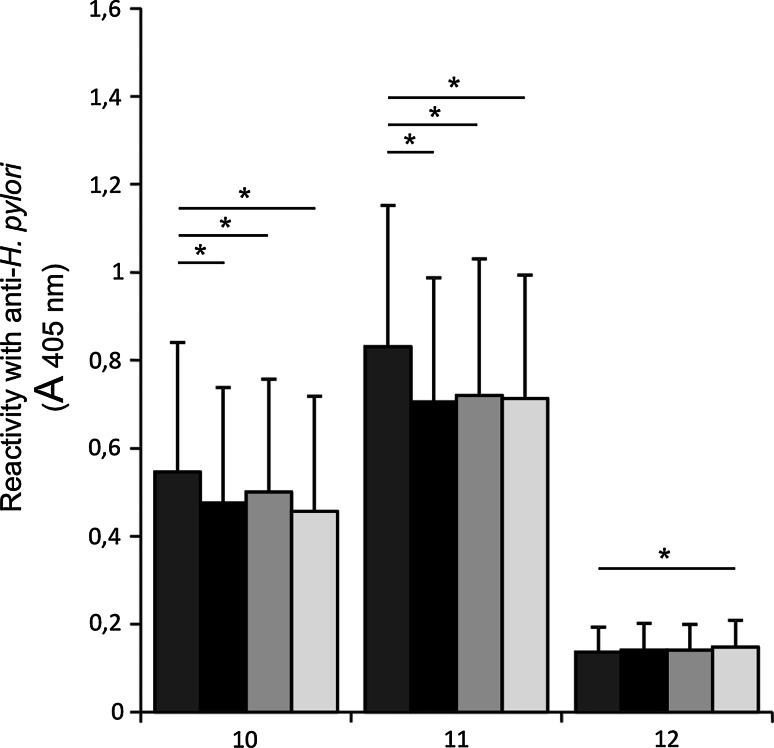
A *babA2*-negative *H. pylori* binding to MUC1 mucin of gastric juices (*n* = 11) selectively captured by anti-MUC1 antibody. MUC1 was not treated (*dark grey bars*), treated with anti-Lewis b (*black bars*), anti-H type 1 (*light grey bars*), or anti-sialyl Lewis x (*white bars*) before addition of *H. pylori* isolates. *10*, *11*, *12*—*babA2*-negative *H. pylori* isolates numbers. *Bars* represent ±SD; * *p* < 0.05

Anti-Lewis b, anti-H type 1, or anti-sialyl Lewis x monoclonal antibodies were added to MUC1 mucin selectively captured by anti-MUC1 monoclonal antibody (ELISA) to determine whether the adhesion activities of *H. pylori* isolates to examined mucin would be affected. In four of *babA2* positive strains (isolates 1, 2, 3, and 4), with high binding to MUC1, clear inhibition of *H. pylori* adhesion after antibodies treatment was observed (Fig. 3). In case of anti-sialyl Lewis x, changes were statistically significant. For anti-Lewis b and anti-H type 1 significance was noted in three mentioned strains. In the other *babA2* positive strains (isolates 5, 6, 7, 8, and 9), with relatively lower level of binding to MUC1, such statistically significant influence of antibody addition was not observed (Fig. 3). In two of three analyzed *babA2* negative strains (strain 10, 11), with high binding to MUC1, inhibition effect after treatment of MUC1 with all examined antibodies was statistically significant. In strain 12 slight increase of binding after antibodies addition was observed with statistical significance for anti-sialyl Lewis x antibody (Fig. 4).

Correlation coefficient values for the relative amounts of Lewis b and sialyl Lewis x on MUC1 versus the level of *H. pylori* bound to MUC1 pretreated or not with proper antibodies are seen in Tables 2 and 3.

**Table 2:** Correlation coefficients calculated for the relative amounts of Lewis b on MUC1 mucin versus the level of *H. pylori* bound to MUC1 pretreated or not with anti-Lewis b

	Strain number	Coefficient value	
		*H. pylori* bound to MUC1	*H. pylori* bound to MUC1 pretreated with anti-Lewis b
Lewis b antigen on MUC1 vs.	1	0.46*	0.42*
	2	0.5*	0.45*
	3	0.13*	0.09*
	4	0.29*	0.26*
	5	0.16	0.23
	6	−0.04	0.04
	7	−0.09	−0.12
	8	0.5*	0.05*
	9	−0.24	−0.36
	10	−0.05*	0.02*
	11	0.06*	0.39*
	12	−0.51	−0.45

Degrees of correlations: *r* = 1—perfect correlation; 0.75 ≤ *r* < 1—high degree of correlation; 0.25 ≤ *r* < 0.75—moderate degree of correlation; 0 < *r* < 0.25—low degree of correlation; −1 ≤ *r* ≤ 0—no correlation. Statistically significant differences (* *p* < 0.05)

**Table 3:** Correlation coefficients calculated for the relative amounts of sialyl Lewis x on MUC1 mucin versus the level of *H. pylori* bound to MUC1 pretreated or not with anti-sialyl Lewis x

	Strain number	Coefficient value (*r*)	
		*H. pylori* bound to MUC1	*H. pylori* bound to MUC1 pretreated with anti-sialyl Lewis x
sialyl Lewis x antigen on MUC1 vs.	1	0.1*	−0.05*
	2	0.03*	0.08*
	3	−0.08*	−0.17*
	4	0.026*	−0.2*
	5	−0.67	−0.66
	6	−0.77	−0.82
	7	−0.22	−0.31
	8	0.07*	−0.05*
	9	−0.12	−0.21
	10	0.32*	0.2*
	11	0.29*	0.26*
	12	0.24	0.24

Degrees of correlations: *r* = 1—perfect correlation; 0.75 ≤ *r* < 1—high degree of correlation; 0.25 ≤ *r* < 0.75—moderate degree of correlation; 0 < *r* < 0.25—low degree of correlation; −1 ≤ *r* ≤ 0—no correlation. Statistically significant differences (* *p* < 0.05)

## Discussion

Interaction of *H. pylori* with specific sugar structures of gastric epithelium is said to be crucial in infection development. Gastric mucins, especially secretory MUC5AC and also epithelial MUC1 can be donors of receptors for bacterial adhesins [1, 15, 16]. MUC1 mucin, closely associated with gastric epithelium is likely to have a special role in *H. pylori* infection development because it can be involved in intracellular signalling [11]. It is suggested that this mucin can act as a kind of releasable decoy [11, 15]. *Helicobacter pylori* binding to MUC1 may initiate signal transduction over the epithelial barrier and cause microbe removal from the luminal surface of epithelium by releasing the extracellular domain of mucin together with attached bacterium [11]. In this way, MUC1 could limit bacterial pathogenesis by preventing against *H. pylori* adherence to epithelial cells. The sugar structures of mucin which are involved in interactions with bacterial adhesins have not been thoroughly characterized so far. Lewis b, H type 1, or sialyl Lewis x are among antigens proposed to be involved in interplays with bacteria [15, 18].

In our work, we decided to use monoclonal antibodies to block these specific antigens on MUC1 which could interact with *H. pylori*. The level of Lewis b and sialyl Lewis x on mucin were assessed (in ELISA tests) with anti-MUC1 (IgG) as a capture antibody and anti-Lewis b and anti-sialyl Lewis x (IgM) as detection ones. The level of H type 1 was not checked because of a lack of IgM class anti-H type 1 detection antibody. Among 12 examined *H. pylori* isolates, 9 were *babA2* positive and 3 were *babA2* negative. However, the presence of BabA adhesins on bacteria was not examined. In some of *babA2* positive strains clear inhibitory effect after using of all examined antibodies was observed. Positive correlations between Lewis b antigen on MUC1 and *H. pylori* binding for these strains additionally support the idea about involvement of this antigen on MUC1 in interaction with the bacterium. However, it is difficult to explain why correlation values in case of pretreatment of MUC1 with anti-Lewis b are still positive (Table 2).

The results for H type 1 antigen are similar (however, without correlations examinations), probably because the same adhesin on *H. pylori* can be responsible for binding of this structure. So H type 1 on MUC1 is also assumed to participate in interplays with *H. pylori.* As inhibitory effect after anti-sialyl Lewis x treatment was also observed, the involvement of sialyl Lewis x antigen in interactions with bacterium can be also suggested, however, *sabA* genotype of examined strains was not examined. Clear inhibitory effect for all used antibodies was also seen in two *babA2* negative isolates. We assume that in this case, antibodies can cause a kind of spherical hindrance and block, to some degree, attachment of microbe to mucin. Because in these *babA2* negative strains the binding with mucin was still observed, our results support the idea about the involvement of other structures on MUC1 in interactions with bacteria.

Interestingly, in the rest of examined strains the effect of antibodies addition was very slight or any (without statistical significance). One possible explanations of this lack of influence of antibodies addition on binding with *H. pylori* is probable existence of other types of interactions occurring between mucins and bacterium. Our assumed inhibition observed not in all patients and lack of demonstration of complete inhibition is in accordance with the results of Clyne and Drumm [21] who confirmed that blocking with monoclonal antibodies for the Lewis b antigen on the gastric epithelium could not totally abolish adherence of *H. pylori.*

Our revealed inhibitory effect is similar to that demonstrated by Osaki et al. [22]. However, the author did not specified exact sugar structures on mucins involved in binding. Apart from that they used IgM class immunoglobulins, while we used both IgG and IgM ones as blocking antibodies. In addition, the authors observed inhibitory effect only when antibodies were added to *H. pylori*. Pretreatment of MKN45 cells with antibodies did not inhibit adhesion of bacteria. Upon these results they suggested that antibodies blocked rather bacterial structures involved in the adhesion system while results of our experiments assume blocking of carbohydrate receptors on MUC1.

It is said that Lewis antigens on LPS of *H. pylori* can be also involved in binding to epithelial structures. Some authors suggest that antibodies can inhibit or increase bacterial adhesion by for example agglutinating bacteria, depending on some properties of antibodies and the presence of Lewis antigens on bacterial LPS. Sheu et al. [10] suggested that IgM isotypes (especially anti-Lewis x antibodies) may promote *H. pylori* adhesion to gastric epithelial cells. The authors assumed the bacterial aggregation and mediation of Le x–Le x interaction (a kind of bridge formation) by examined antibody, with participation of Lewis x on both *H. pylori* LPS and gastric epithelium [10, 23]. However, in our study we did not observed special enhancement of bacterial adhesion to MUC1 in strains with high level of Lewis b in comparison with strains with lower level of this antigen (strain 2 and 4; Figs. 1, 3). So upon these results we rather can not suggest participation of Lewis b of bacterial LPS in adhesion to MUC1. Additional experiments should be performed.

Summarizing, our results confirmed probable participation of Lewis b, H type 1, and also sialyl Lewis x of MUC1 mucin in interactions with *H. pylori* independently on *babA* genopositivity. Not full inhibition and a lack of this effect in some strains suggest an existence of other mechanisms of *H. pylori* adherence to mucin.
